# Survey and critical appraisal of pharmacological agents with potential thermo-modulatory properties in the context of artificially induced hypometabolism

**Published:** 2015-07-19

**Authors:** Marcel C. Dirkes, Thomas M. van Gulik, Michal Heger

**Affiliations:** 1 Department of Experimental Surgery, Academic Medical Center, University of Amsterdam, Amsterdam, the Netherlands; 2 Current affiliation: Philips Research, Eindhoven, the Netherlands

**Keywords:** thermoneutral zone, hypothermia induction, pharmacological agents, animals, body temperature

## Abstract

A reduction in body temperature can be achieved by a downward adjustment of the termoneutral zone, a process also described as anapyrexia. Pharmacological induction of anapyrexia could enable numerous applications in medicine. However, little is known about the potential of pharmacological agents to induce anapyrexic signaling. Therefore, a review of literature was performed and over a thousand pharmacologically active compounds were analyzed for their ability to induce anapyrexia in animals. Based on this analysis, eight agents (helium, dimethyl sulfoxide, reserpine, (oxo)tremorine, pentobarbital, (chlor) promazine, insulin, and acetaminophen) were identified as potential anapyrexia-inducing compounds and discussed in detail. The translational pitfalls were also addressed for each candidate compound. Of the agents that were discussed, reserpine, (oxo)tremorine, and (chlor) promazine may possess true anapyrexic properties based on their ability to either affect the thermoneutral zone or its effectors and facilitate hypothermic signaling. However, these properties are currently not unequivocal and warrant further examination in the context of artificially-induced hypometabolism.

## 1. Introduction

**Figure 1.**
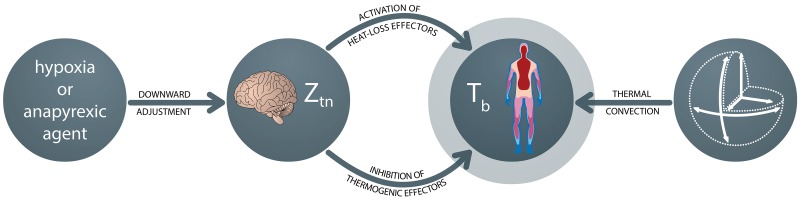
Regulation of body temperature (T_b_) through change of the thermoneutral zone (Z_tn_). The first sphere on the left indicates an initial external trigger, which may be an environmental stimulus such as hypoxia or a pharmacological agent with anapyrexic properties. These triggers can lead to a downward adjustment of the Z_tn_ (second sphere). In turn, the reduction of the Z_tn_ leads to activation of heat loss mechanisms (sweating, behavioral adaptation, panting, vasodilation) and inhibition of thermogenesis (shivering, activation of brown adipose tissue, behavioral adaptation, vasoconstriction, piloerection), resulting in a reduction of T_b_ (third sphere). The extent of T_b_ reduction is dependent on the rate of thermal convection, which in turn is dependent on the body surface:volume ratio (fourth sphere).

An organism’s core body temperature (T_b_) is of key importance to its physiological function, as reflected by the meticulous regulation of T_b_. The plasticity of thermal regulation is demonstrated by numerous pathological conditions, such as the increase in T_b_ (pyrexia) during an infection. A lesser known, but potentially equally important thermal adaptation mechanism, is regulated decrease in T_b_ (anapyrexia). Anapyrexia can be described as the opposite of fever, namely a lowering of the boundaries between which the body considers itself thermoneutral (the thermoneutral zone, Z_tn_), and concurs with the inhibition of thermogenic processes and the activation of heat loss mechanisms (Figure 1).

The ability to lower the Z_tn_ is an established feature of poikilothermic animals, one that is only starting to be recognized in homeothermic animals [1]. The integration and processing of thermoregulatory signals is believed to involve several intricate neural pathways encompassing both peripheral sensory neurons and central hypothalamic neurons and nuclei [2-5], including the preoptic anterior hypothalamus (POAH) [3, 4, 6-8]. Together these pathways manage the Z_tn_, which in turn manages thermogenesis (e.g., shivering, activation of brown adipose tissue (BAT), vasoconstriction, tachycardia, tachypnea, piloerection, and behavioral accommodation) and heat loss (e.g., vasodilation, sweating, panting, and changes in behavioral patterns such as the pursuit of lower environmental temperatures (T_a_)) (Figure 1) [8,9]. Readers interested in the neuroanatomical networks that govern mammalian thermoregulation via the POAH are referred to a panel of excellent papers by Morrison and Nakamura on this subject [10-13].

A method to induce anapyrexia in small animals is by subjecting the animals to hypoxia, which triggers regulated hypothermia and corollary hypometabolism in some species as a countermeasure against the hypoxic, and thus potentially lethal, conditions [14-17]. One of the putative regulatory mechanisms is centered on carotid body sensing [18, 19]. Carotid bodies are clusters of chemoreceptors and cells near the bifurcation of the carotid artery that detect changes in oxygenation-related parameters, including partial pressure of oxygen and carbon dioxide as well as pH and temperature [20, 21]. When hypoxia is sensed, anapyrexia is induced through the inhibition of thermogenic effectors and activation of cooling effectors [1,16,22-24], which are under control of the POAH [7, 8,11,25]. This protective mechanism (Figure 1) is believed to be rooted in evolution, and there is evidence that such a mechanism is preserved in man, at least to an extent [14, 26]. However, hypoxia is generally not employed in the clinical setting as a patient’s already compromised state may be exacerbated at low oxygen tensions. Nevertheless, the artificial modulation of a patient’s Z_tn_ is of great interest because of the protective effects that are associated with hypometabolism [17].

Inasmuch as artificial (clinical) regulation of anapyrexia via the hypoxia-Z_tn_ axis does not constitute the most suitable and practical means, alternative methods have been explored. One interesting and potentially viable approach is pharmacological modulation of thermoregulatory cold receptors in the skin [27, 28]. Studies published by Andrej Romanovsky’s Fever Lab have demonstrated that selective inhibition of the transient receptor potential melastatin-8 (TRPM8) channel (cutaneous cold receptor) with M8-B effectively decreases the T_b_ in mice and rats via several thermal effectors (thermopreferential behavior, tailskin vasoconstriction, and brown adipose tissue) [27]. Alternative strategies aimed at gaining control over the Z_tn_ by pharmacological means at intervention sites other than cutaneous cold receptors also appear promising and encompass several proposed compounds such as arginine, vasopressin, lactate, adenosine, histamine, delta- and kappa opioids, nitrogen monoxide, and carbon monoxide [1]. Although these agents harness potential for clinical application, most of the compounds are associated with undesired sideeffects that have confined their use to the experimental setting.

The pharmacological induction of hypothermia through modulation of the Z_tn_ has proven quite difficult in practice, particularly since the number of reports on methods to induce anapyrexia is limited. Nevertheless, the downward adjustment of the Z_tn_ by pharmacological agents may have numerous beneficial implications for medicine and biotechnology, but also for sports and aviation/space travel. Consequently, the identification of new anapyrexic agents or re-evaluation of established compounds for their anapyrexic properties has become increasingly important. Subjecting all known pharmacological agents to specific empirical investigations, however, would be exhaustive and comprehensive.

## 2. Aim of the study

In order to provide an accessible summary of potentially useful pharmacological agents for the induction of anapyrexic signaling, we performed a review of literature and analyzed over a thousand pharmacologically active compounds for their ability to induce anapyrexia in animals. The most viable candidates were identified on the basis of the magnitude of the reported heat loss and critically appraised in the context of the Z_tn_-mediated heat loss mechanisms (Figure 1). In this study we focused specifically on the most studied compounds that potentially harness anapyrexic properties and addressed the candidate drugs against a backdrop of empirical evidence related to mainly pharmacodynamics and toxicology. The secondary purpose of this review was to guide novel research with ‘old compounds’ in the context of anapyrexic signaling by elaborating on the discrepancies in reported data and knowledge gaps. Subsequent reviews will focus on the physiological, biochemical, and neurological mechanisms of anapyrexic signaling in terms of hypometabolism-inducing pharmacological agents (manuscript submitted) and the role of hypoxic sensing via e.g., carotid and aortic bodies with respect to POAH- mediated thermoregulation (manuscript in preparation).

## 3. Visualizing drug-induced changes in body temperature

**Figure 2.**
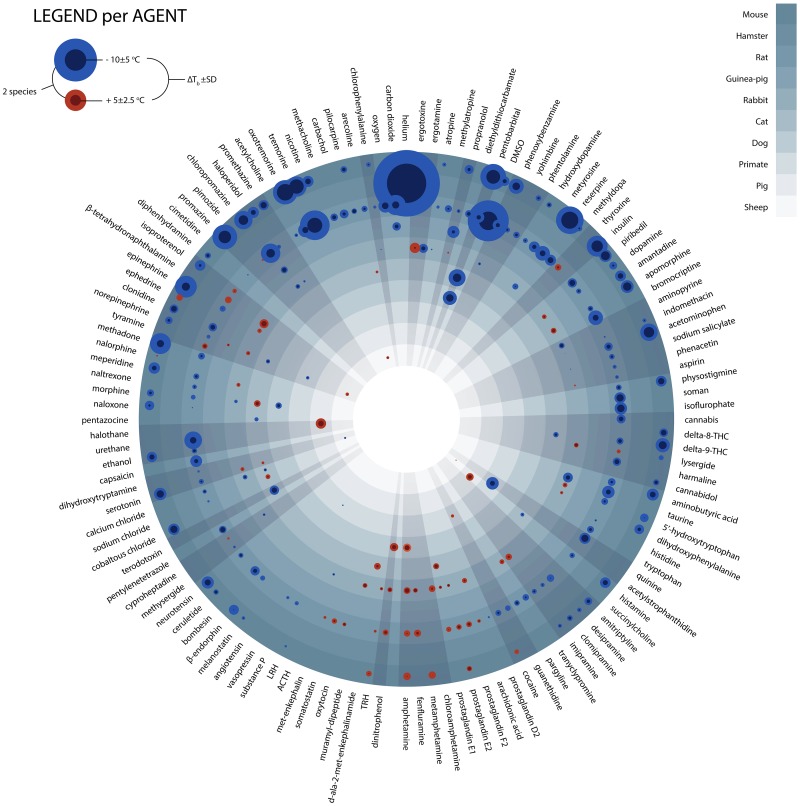
Change in T_b_ upon exposure to pharmacological agents. All presented data are derived from reviews published by Clark *et al*. [29-36]. These reviews total 18,808 reports on changes in T_b_ (ΔT_b_) following exposure to a biochemical agent. All avian (628 reports), aquatic (46 reports), reptilian (31 reports), and naturally hibernating species (164 reports) were excluded on the basis that they are intrinsically endowed with different mechanisms regarding thermoregulation and hypometabolism [37]. All reports of human ΔT_b_ (1,285 reports) were excluded on the basis that they are not likely to be performed under standardized or controlled circumstances. All reports including a pre-existing febrile state (2,680 reports) were excluded on the rationale that these do not reflect an effect on healthy individuals and possibly only affect an increased Z_tn_. All reports with no quantitative data were excluded (6,591 reports). In case of multiple data points, the largest ΔT_b_ was included. To improve the validity of T_b_ values, all agents that had < 10 reports within one species were also excluded. The final dataset, consisting of ≥10 reports/agent/species, was used for analysis and visualization. Data analysis was performed in Matlab R2011a (Mathworks) and graphically processed in Adobe InDesign CS5 (Adobe). The ΔT_b_s are plotted as bicolored spheres, whereby cooling is indicated in blue and heating in red. The mean ΔT_b_ of each agent per species (n ≥ 10) is represented by the inner diameter of the sphere. The difference between the inner and outer diameter of the sphere represents the standard deviation. All spheres are projected against a layered concentric background, whereby each layer (separated by different shades of gray) represents a species as indicated in the legend (upper right). The agents are grouped according to the classification used in the original manuscripts, delineated by the outward radiating gray areas. For better viewing, the figure is also provided online as supplemental Figure 1.

Between 1979 and 1986 eight extensive reviews on changes in T_b_ after exposure to pharmacological agents were published by Wesley G . Clark in Neuroscience and Biobehavioral Reviews [29-36]. According to the author, “this survey … intended to provide an immediate source of information on drug-induced changes in thermoregulation” [31]. Published prior to the coining of the term ‘anapyrexia,’ the reviews furnish relevant information on 1,295 agents in 48 mammalian species, although the size of the data compilation makes it difficult to effectively assess the anapyrexic potential of all agents. Therefore, we have created a visual tool to assist in the analysis of data by plotting the T_b_ change per compound and per species in a single diagram (Figure 2, see legend for meth- ods). In short, a blue sphere indicates a reduction in T_b_, which reflects the inability to maintain thermal homeostasis and hence points to potential anapyrexic properties of the agent. Contrastingly, a red sphere indicates an increase in T_b_ and thus pyretic properties of the agent. The size of the sphere is proportional to the magnitude of the change in T_b_. Before elaborating on the most promising anapyrexia-inducing agents, it is important to outline some limitations of the analysis.

## 4. Limitations of the analytical method

The data in Figure 2 have four important limitations. Firstly, although each data point (sphere) represents the average of ≥ 10 reports, the number of animals in each report was not taken into account simply because the sample size was not indicated in every report, making a weighted analysis impossible. This may skew the data insofar as the result from one animal bears equal weight as the mean of results acquired with a larger group size.

Secondly, the change in T_b_ was not corrected for the T_a_, which can have a considerable effect on convection and therefore the measured depth of hypothermia, particularly in small species [38]. Although most experiments were conducted at an average T_a_ that was within a normally accepted range (~18-25 °C), a few exceptions must be noted, such as for helium and DMSO, where the experiments were performed at an average T_a_ of 10.0 ± 13.3 °C and 12.5 ± 10.3 °C, respectively. The complete list of T_a_s and the quantitative data of Figure 2 can be found in supplemental Table S1.

**Table S1:**
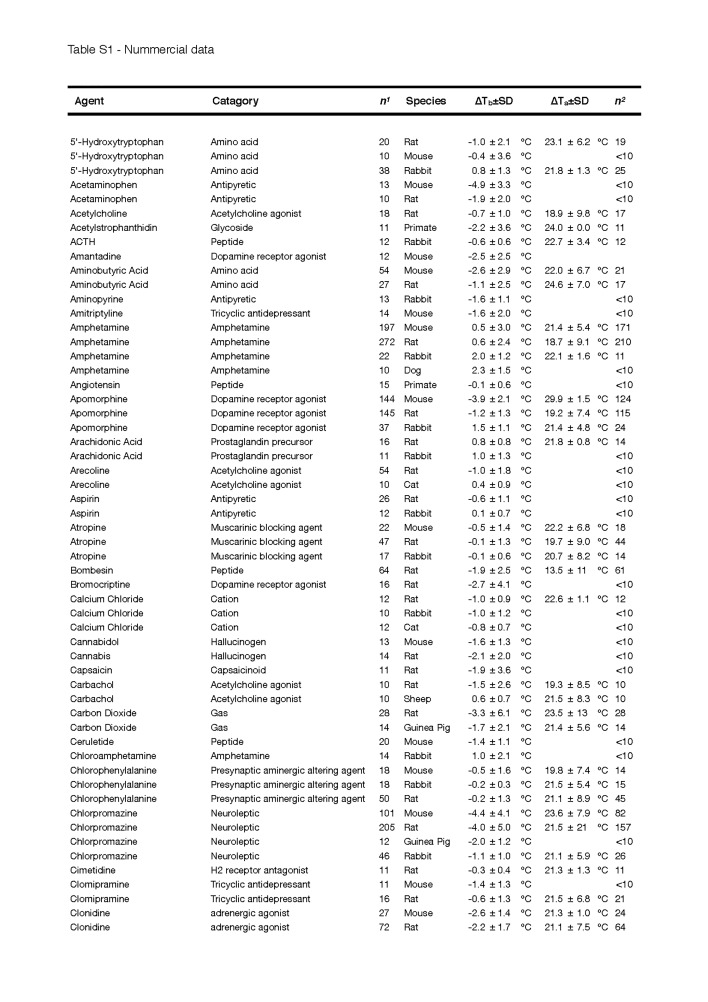
Nummercial data

**Table S1:**
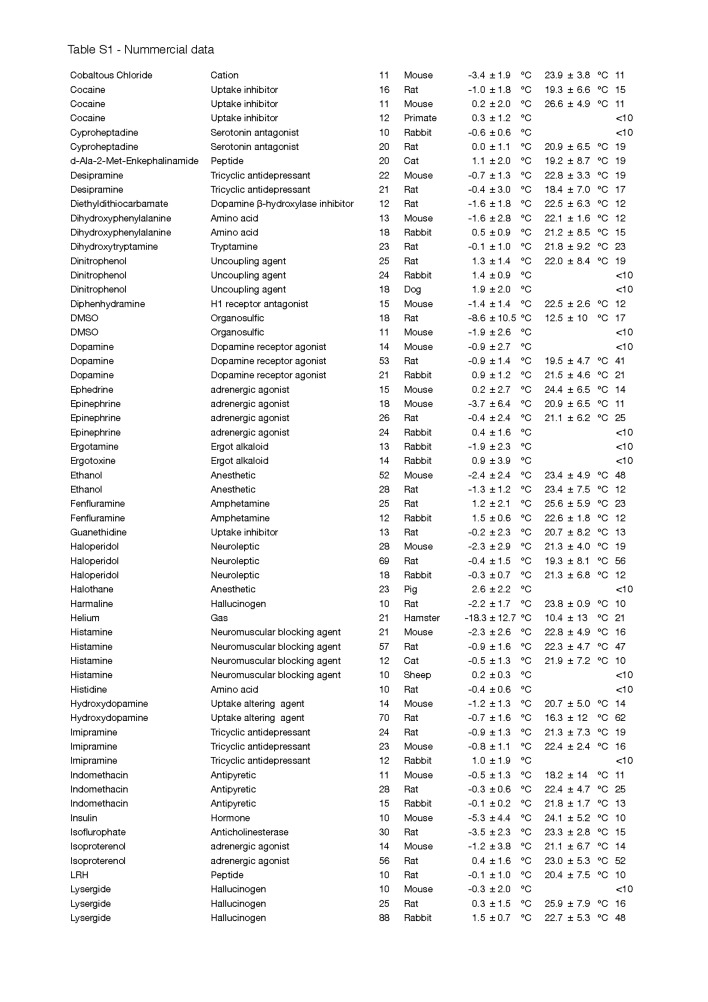
Nummercial data

**Table S1:**
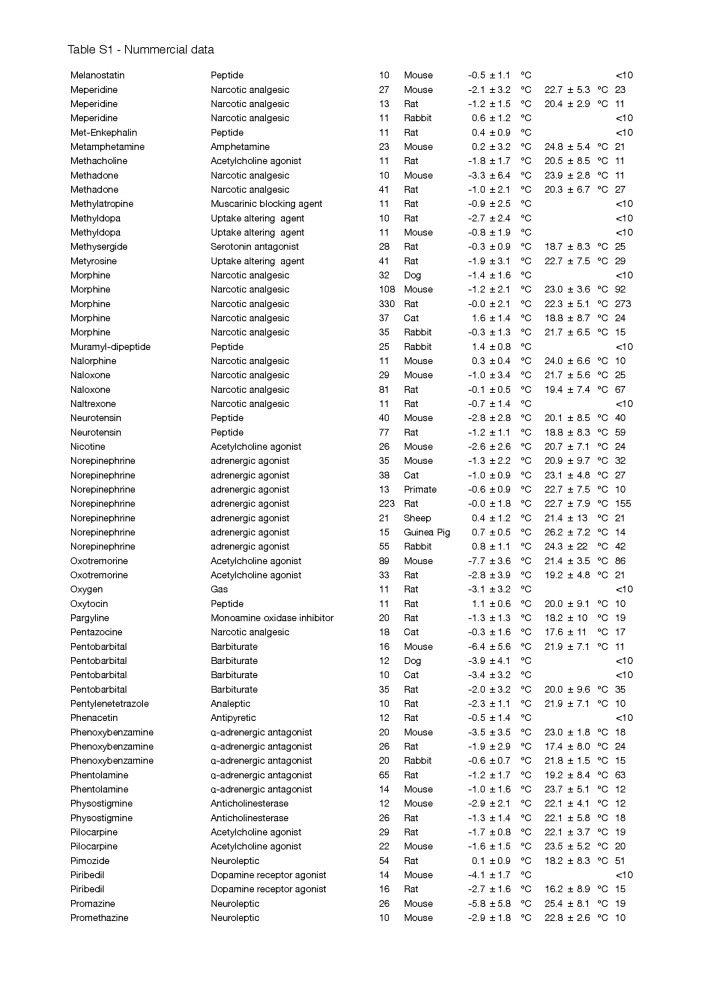
Nummercial data

**Table S1:**
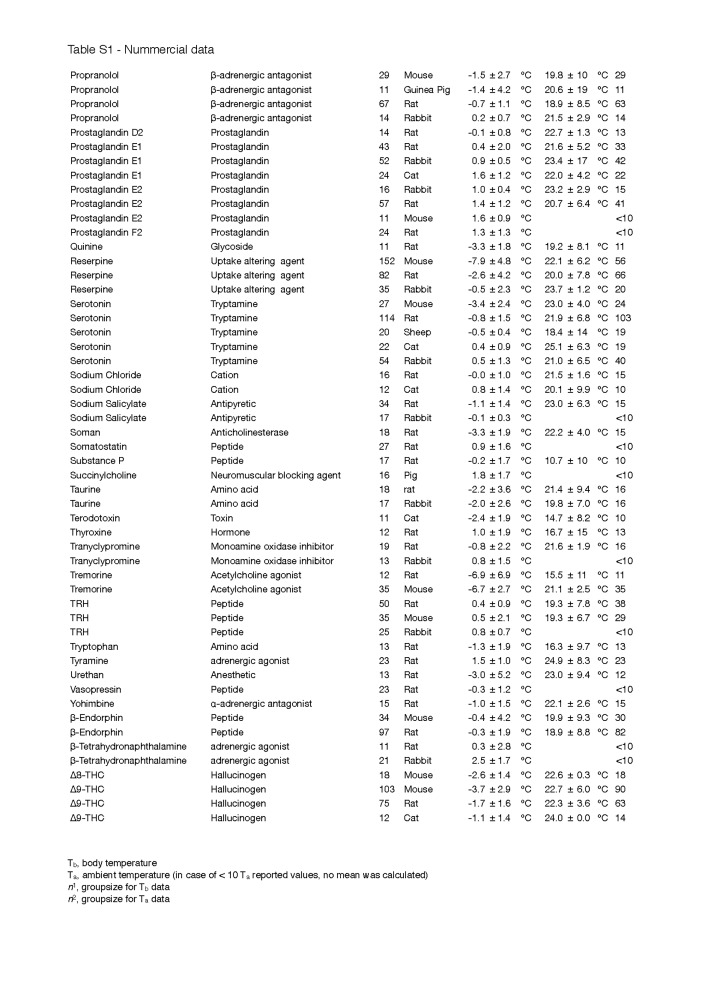
Nummercial data

Thirdly, the dosage and administration route, which can affect the disposition of a pharmacological agent, were not accounted for in the analysis. An example of a dose-dependent effect on T_b_ can be seen with morphine (Figure 2). Morphine has the potential to both increase (cat) and decrease (dog) the T_b_. These opposite findings may originate from differences in dosing since low concentrations of morphine (≤ 5 mg/kg intravenous bolus) cause a rise in T_b_, whereas high concentrations (≥ 10 mg/kg intravenous bolus) cause a marked decrease in T_b_ [39].

Lastly, the manner in which experiments were performed was discounted. The reports from which the data were collected were published between 1979 to 1987, i.e., just after the introduction of ‘good laboratory practice’ criteria in the late 1970’s [40]. This may have had an impact on the accuracy of the obtained and published results.

## 5. Thermal convection: the importance of surface:volume ratio and metabolic rate

**Figure 3.**
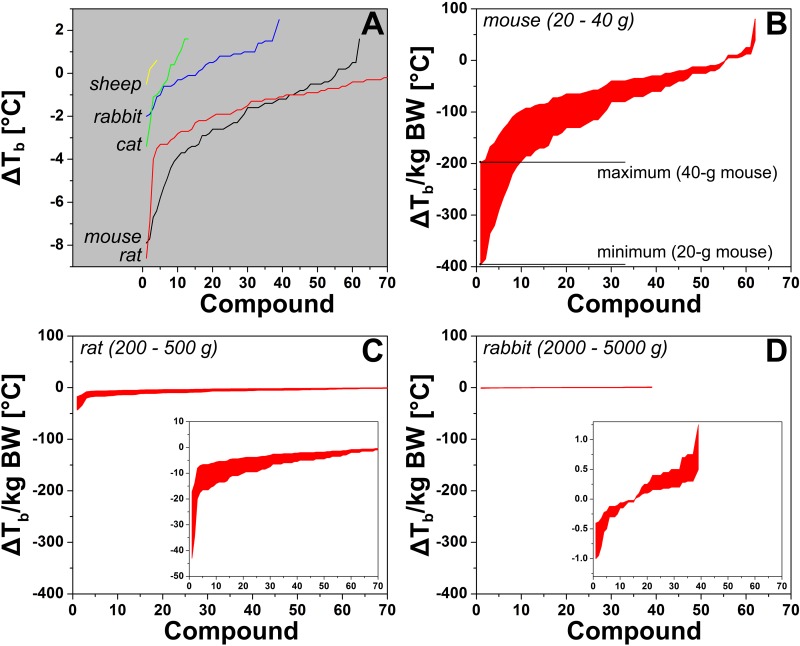
(A) Change in core body temperature (ΔT_b_) per species plotted as a function of compound with the most profound effect on T_b_ (1, *x*-axis) to the compound with the least effect on T_b_ (up to 70, *x*-axis). The ΔT_b_ represents the mean of all ΔT_b_s reported for the respective compound in the respective species that were included in the analysis. Research data were included on the basis of the criteria described in section 3 and the legend of Figure 2. The complete data set containing all the species is provided in Table S3. The data were normalized to the maximum and minimum common body weights (BW) of laboratory mice (B), rats (C), and rabbits (D) and should be read vertically per compound, whereby the upper limit is the minimum ΔT_b_ for the heaviest animals. The actual recorded values fall between the upper and lower bounds per compound. Note the different *y*-axis scaling of the inset plots. Common body weights were obtained from the internet (e.g., laboratory animal providers such as Harlan and Charles River).

In addition to the evident thermomodulatory effects induced by some of the compounds, the data in Figure 2 is subject to two important principles that may affect anapyrexia, namely the body surface:volume ratio and Kleiber’s law (discussed in [38]). With respect to the former, small animals cool down at a faster rate than larger animals because their relatively large surface:volume ratio facilitates more extensive heat exchange with the environment (Figure 1). Corroboratively, the largest changes in T_b_ are found in mice and rats (Figure 3A). Kleiber’s law is an allometric law that describes an inverse relationship between metabolic rate and body size [41]. One of the pillars of this law is that smaller species need proportionally more energy than larger species to sustain their metabolism. Both principles essentially dictate that a reduction in Z_tn_ can manifest itself faster and more profoundly in small species compared to larger species. Unfortunately, the distribution of the data in Figure 2 is slightly biased towards the smaller species (supplemental Table S2), which clouds the unequivocal manifestation of these principles across all species included in the analysis.

**Table S2:**
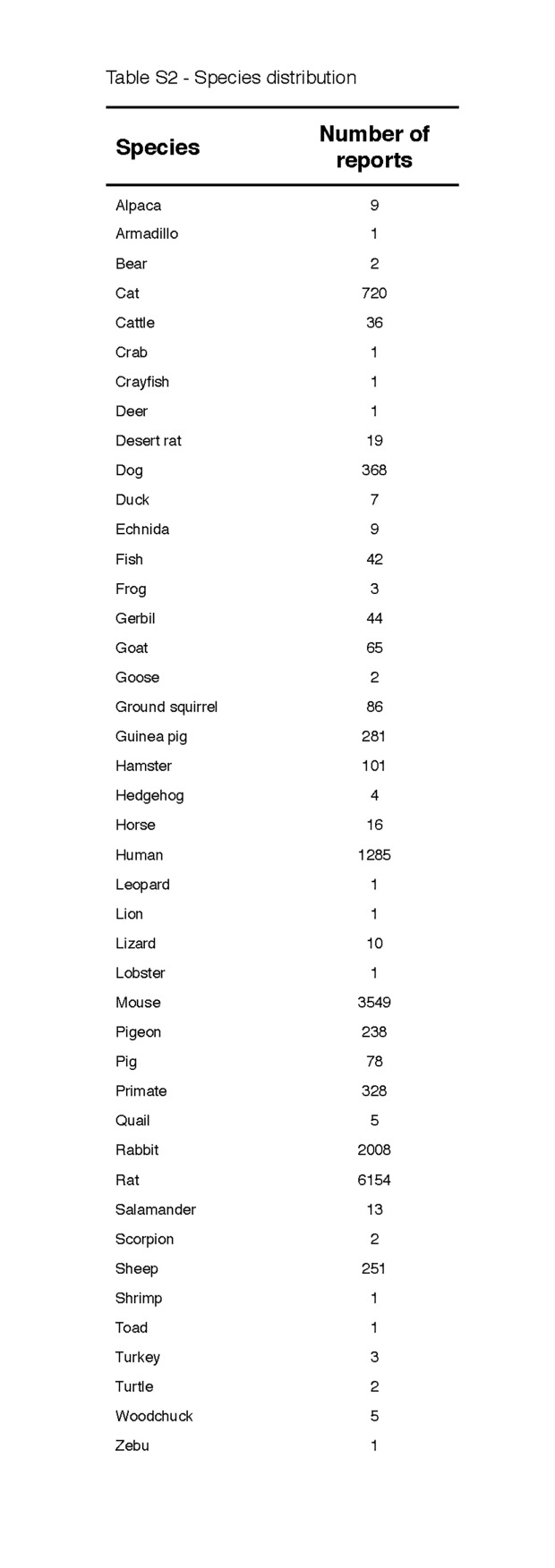
Species distribution

Nevertheless, a good illustration can be provided on the basis of mice (N = 62), rats (N = 96), and rabbits (N = 39) alone, as shown in Figure 3B-D, where the change in T_b_ was organized from greatest to lowest and plotted per compound following normalization to body weight (in kg). The upper limit and lower limit weights of these laboratory animals were used to demarcate the maximum and minimum boundaries of the T_b_ change per unit weight. This was done to semi-standardize the data because a considerable fraction of the articles from which the data were derived did not report the mean body weight or weight range of the animals used in the experiments. When normalized to body weight, the heat loss per kg body weight is most sizeable in mice and smallest in rabbits, confirming the observations in Figure 2 and clearly illustrating both principles described above. A complete list with the categorized variables is provided in Table S3.

## 6. Compounds with anapyrexic potential

**Figure 4.**
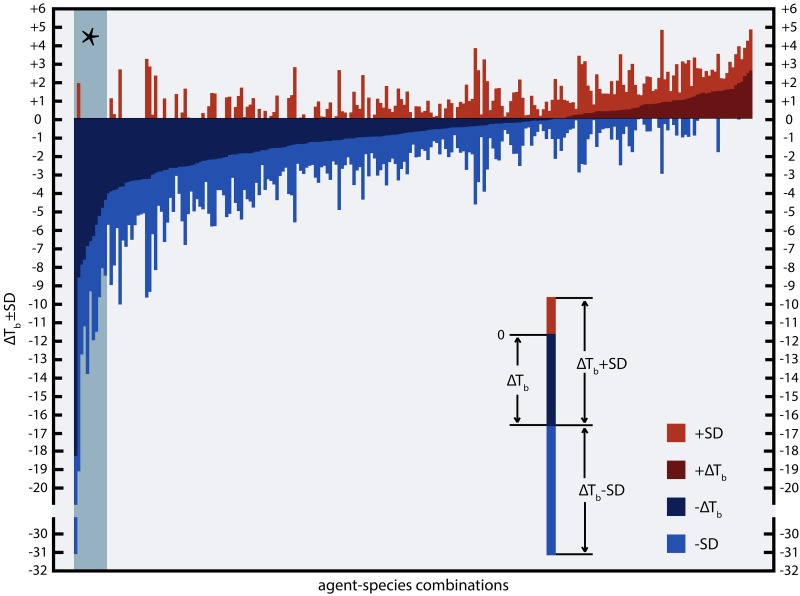
Ranked effect size of the change in T_b_ following exposure of an animal to a pharmacological agent. For the source of the data and inclusion criteria see the legend of Figure 2. All agent-species combinations (*x*-axis) were ranked by mean effect size (*y*-axis) with standard deviations (see legend within panel). The section marked with an asterix (*, top left) marks the agents that are discussed in more detail.

To identify anapyrexic agents, the data in Figure 2 were plotted according to the magnitude of T_b_ change, from the greatest decrease to the greatest increase in T_b_ (Figure 4). The magnitude of T_b_ decrease was used as the standard parameter to gauge anapyrexic signaling potential insofar as a downward modulation of T_b_ is the most important hallmark of anapyrexic signaling and does not occur in hibernators and non-hibernators in the absence of a Z_tn_ adjustment under non-stimulatory circumstances (e.g., under conditions of normoxia, abundant food supply, T_a_ ≈ T_b_, etc.) [37-42]. Based on this figure, eight agents (eleven agent-species combination) with the largest T_b_ decrease were selected as the most promising agents in terms of anapyrexic potential. These agents include helium (hamster, ΔT_b_ = −18.0 ± 12.7 °C), dimethyl sulfoxide (DMSO, rat, ΔT_b_ = −8.6 ± 10.5 °C), reserpine (mouse, ΔT_b_ = −7.9 ± 4.8 °C), (oxo)tremorine (oxotremorine, mouse, ΔT_b_ = −7.7 ± 3.6 °C; tremorine, rat, ΔT_b_ = −6.9 ± 6.9 °C, mouse, ΔT_b_ = −6.7 ± 2.7 °C), pentobarbital (mouse, ΔT_b_ = −6.4 ± 5.6 °C), (chlor) promazine (chlorpromazine, mouse, ΔT_b_ = −4.4 ± 4.1 °C; promazine, mouse, ΔT_b_ = −5.8 ± 5.8 °C), insulin (mouse, ΔT_b_ = −5.3 ± 4.4 °C), and acetaminophen (mouse, ΔT_b_ = −4.9 ± 3.3 °C).

The observed reduction in T_b_ for these agents raises an important question: should the observed T_b_ reduction be diagnosed as anapyrexia or hypothermia, their sole difference being that the decrease in T_b_ is the result of a downward adjustment of the Z_tn_ in case of anapyrexia? A direct measurement of the Z_tn_ following administration of an agent would constitute the ultimate method to determine anapyrexic potential. However, due to the fact that we currently neither fully understand the body’s temperature integration system nor have the means to monitor it, direct measurement of the boundaries that make up the Z_tn_ is impossible. Consequently, the gold standard in determination whether an organism is within the Z_tn_ boundaries is based on the activity of thermal effectors (Figure 1).

Therefore, in the next sections the eight most promising agents are addressed in the context of their effect on thermogenic and heat loss effectors such as shivering, BAT activity, sweating, vasoconstriction/vasodilation, and behavioral accommodation (Figure 1).

### 6.1. Helium

Helium is a noble gas with minimal direct biological activity [43]. Due to its biological inertness it is unlikely that helium induces cooling (Figure 2) via direct impact on the Z_tn_. Instead, the thermoregulatory mechanism of helium may be based on its ability to augment the rate of heat convection.

The thermal conductivity of helium is 5.75 times higher than that of nitrogen [44]. When inhaling a gas mixture in which nitrogen is replaced by helium (e.g., Helox), the increased thermal conductivity of helium can accelerate changes in T_b_ in a proportional manner to T_a_. The efficiency with which the pulmonary vascular bed is able to exchange heat is reflected by the clinical use of helium gas as a tool to accelerate the rewarming of hypothermic patients in combination with regular heating therapy [45]. An important prerequisite for helium-mediated reduction in T_b_, as shown in Figure 2, is that it must be accompanied by a low T_a_, which indeed averaged 10.0 ± 13.3 °C (supplemental Table S1).

Accelerated heat convection as the mechanism behind helium-induced hypothermia is further supported by the absence of change in behavioral thermoregulation in animals. In mice exposed to normoxic normobaric helium, there is no increase in heat avoidance, suggesting that helium does not have an effect on the Z_tn_ [46]. The mechanism of helium-induced hypothermia is therefore ascribable to its biophysical rather than its biochemical properties.

### 6.2. Dimethyl sulfoxide

DMSO has been widely exploited as an analgesic, anti-inflammatory agent, cryoprotectant, radioprotectant, transcutaneous transporter, barbiturates enhancer, and organ preservative [47-50]. However, the clinical use of DMSO was banned in 1965 by the Food and Drug Administration, restricting its applicability to experimental use. In the experimental setting the hypothermic effects of DMSO are markedly evident without inducing notable arterial and neuronal damage following systemic administration [51], suggesting that DMSO may be an anapyrexic agent.

In rats, exposure to DMSO resulted in a decreased T_b_ with a simultaneous decrease in oxygen consumption and respiratory quotient, which are characteristic of a hypometabolic state [52]. The reduction in metabolism was likely triggered by a reduction in Z_tn_, as a sustained Z_tn_ would initiate a temporary increase in oxygen consumption (a thermogenic effector mechanism) in an attempt to bring the T_b_ back to a thermoneutral level.

Besides anapyrexic effects, it has been suggested that DMSO has the potential to act via the thyroid gland. Normally, the thyroid gland is under hypothalamic control to produce metabolism-promoting thyronines, which require iodine (I) for their synthesis. Upon exposure of mice to DMSO via intraperitoneal injection, the uptake of ^131^I was shown to be attenuated, suggesting that the mechanism of DMSO-induced hypometabolism may be co-regulated by the thyroid gland [52]. However, it cannot be excluded that the observed dose-dependent reduction in ^131^I uptake was a result of DMSO-induced reduction in T_b_, or that it originated from a change in hypothalamic control (e.g., due to changes in Z_tn_).

Nevertheless, the question remains whether the decline in T_b_ is anapyrexic in nature or mediated by peripheral factors. In an experiment focused on behavioral thermoregulation in rats, exposure to DMSO, which had a lowering effect on T_b_, led to a 2-fold increase in the need for external heat reinforcement [53]. This finding is in direct contradiction to anapyrexia, in which the lowered T_b_ would manifest itself by heat avoidance rather than reinforcement. In addition, the experiments performed in rats were performed in a temperature range of 0-26 °C [32]. The observation that DMSO caused such a large change in T_b_ in a small species such as a rat (Figure 3A, C) may in part be explained by the heavy cold thermal load. Finally, the interaction between DMSO and H_2_O is exothermic in nature, causing erythema when applied on the skin [48]. In spite of the initial warm sensation, the accompanying vasodilation can considerably augment the rate of heat loss, which may be further exacerbated by a DMSO-induced decrease in shivering [52].

It therefore appears that the physiological effects of DMSO do not completely fit the physiological response profile of an anapyrexic agent.

### 6.3. Reserpine

Reserpine is prescribed to patients as an antipsychotic or anti-hypertensive drug. In the experimental setting the drug is often avoided because it induces hypothermia in small animals (Figure 2).

The mechanism of reserpine-mediated hypothermia is supposedly based on massive depletion of monoamines. Experiments in which cerebral monoamine concentrations were measured following reserpine administration revealed that reserpine depletes dopamine, norepinephrine, and serotonin levels [54, 55]. More specifically, reserpine was shown to decrease monoamine levels in the hypothalamus, an important part of the thermoregulatory system, suggesting anapyrexic potential [56].

Several studies have attempted to narrow down specific monoamines that may be involved in reserpine-induced hypothermia. These demonstrated that the reduction in cerebral serotonin levels by para-chlorophynylalanine was not accompanied by hypothermia [54]. Similarly, no hypothermic response was observed following a reduction in cerebral dopamine and norepinephrine levels by α-methyl-*m*-tyrosine [54].

However, exposure of reserpine-induced hypothermic mice to SKF-38393, a D_1_-like (dopamine) receptor agonist, led to a significant reversal of hypothermia [57]. Subsequent addition of SCH-23390, a D_1_-like receptor antagonist, abrogated the T_b_ raising effects of SKF-38393, suggesting a central role for the D_1_-like receptor in reserpine-induced hypothermia [57]. On the other hand, apomorphine, a non-specific dopamine receptor agonist, had the ability to induce hypothermia to a similar depth as reserpine, altogether indicating that the dopamine receptor plays an ambivalent but prominent role in the observed hypothermia following reserpine exposure [58].

The inconsistencies in reserpine-induced hypothermia mechanisms have been the focus of numerous reports [59]. However, most reports predominantly address augmentation of monoamines and their associated receptors of the central nervous system, while only a few reports describe the effects on (peripheral) thermogenic effectors. Reserpine has important inhibitory effects on thermogenic effectors such as BAT, where it has been shown to deplete norepinephrine stores [60]. As BAT is under adrenergic control, depleted norepinephrine stores can lead to severely impaired thermogenic activity [61].

Considering the current knowledge on reserpine, it could be postulated that reserpine constitutes the proverbial cannon to kill a mosquito. Generally, monoamine depletion causes many depressive effects that may encompass the POAH, and may therefore cause anapyrexia by a direct effect on the Z_tn_. Irrespective of the central effects, reserpine’s inhibitory effect on BAT will promote hypothermia indirectly by its inhibitory effect on thermogenesis through local norepinephrine depletion. These favorable properties notwithstanding, more specific knowledge on reserpine’s mechanism of action is required, particularly with respect to central effects, before it can be categorized as a legitimate anapyrexic agent and clinically implemented as such.

### 6.4. (Oxo)tremorine

The use of tremorine and its metabolite oxotremorine are limited to the experimental setting. Exposure to tremorine does not only lead to hypothermia, but also induces generalized tremor and rigidity, owing to its muscarinic acetylcholine transport agonism [62].

Induction of hypothermia with (oxo)tremorine coincides with an important anapyrexic-like change in thermoregulatory behavior in animals, namely the active search for a cooler environment [63, 64]. This effect can be readily reversed by addition of atropine, a muscarinic acetylcholine receptor antagonist, suggesting involvement of cholinergic receptors in the management of Z_tn_ and T_b_ [63]. Further evidence supporting the anapyrexic effect of (oxo)tremorine is the profound hypothermia upon injection of tremorine directly into the POAH, a regulatory site involved in management of the Z_tn_. A hypothermic response is absent when tremorine is injected into other cerebral regions [65]. Cholinergic receptors are putatively associated with T_b_ control. However, as they have been shown to trigger both pyretic and anapyrexic responses, their exact role in T_b_ control remains unclear [66-68].

Contrary to the central anapyrexia-like effects of (oxo)tremorine, the effects on peripheral effectors are equivocal. In larger animals, exposure to oxotremorine produces shivering, vasoconstriction, and signs of inhibited panting, altogether culminating in a T_b_ increase [69, 70]. Although the vasoconstrictive response does not necessarily imply pyretic signaling but rather an effect of low blood pressure associated with oxotremorine exposure, the shivering can be associated with pyrexia or constitute a local effect [71]. The increase in T_b_ could possibly be supported by BAT activity, albeit the direct effects of (oxo)tremorine on BAT are largely unexplored. However, due to the limited presence of cholinergic fibers in BAT and its predominantly adrenergic control, BAT most likely plays no role in the (oxo)tremorine-induced thermogenesis [61, 72].

The capacity of (oxo)tremorine to induce both anapyrexia (i.e., thermoregulatory behavioral patterns, POAH-specific effects) and pyrexia (i.e., shivering, inhibition of panting) gives rise to the clinically relevant question whether its pyretic responses are centrally or peripherally regulated. The emphasis on the underlying mechanism of the pyretic responses is related to the possibility to control one of the thermogenic effectors, namely shivering, by the use of muscle relaxants. If shivering accounts for the majority of thermogenesis after (oxo)tremorine administration, anapyrexia could be effectively induced by (oxo)tremorine during hypothermic surgery on the condition it is co-administered with muscle relaxants to suppress the shivering.

Unfortunately, the amount of data on the peripheral mechanisms of (oxo)tremorine is very limited. To assess the anapyrexic potential of (oxo)tremorine, its mechanism on peripheral thermal effectors must be elucidated first.

### 6.5. Pentobarbital

Pentobarbital-induced hypothermia is believed to be facilitated by an increase in heat loss via dilation of cutaneous blood vessels [73]. The lack of Z_tn_ involvement in pentobarbital-induced hypothermia is supported by studies in rats, where no hypothermic response was observed following injection of pentobarbital into the POAH [73, 74]. However, intracerebroventricular injection of a 6-fold higher pentobarbital concentration resulted in a T_a_-dependent hypothermic response that was accompanied by cutaneous vasodilation [75]. Accordingly, the T_a_-dependent decrease in T_b_ and vasodilation imply that high intracerebroventricular pentobarbital concentrations produce systemic triggers that result in hypothermia, but not via the Z_tn_. The absence of an anapyrexic effect is further supported by the lack of changes in thermoregulatory behavior in mice and rats [76].

Despite the widespread view that pentobarbital has no anapyrexic effects, more recent studies suggest that γ-aminobutyric acid (GABA) receptors, the main target of pentobarbital, may play a role in thermal homeostasis [77]. An *in vitro* study using hypothalamic medial preoptic slices revealed that both GABA_A_ and GABA_B_ receptor agonists inhibit neuronal tonic activity, implying a potential of GABA receptor agonists to modulate the Z_tn_ [78]. In murine GABA_B_ knockout and partial knockdown models, hypothermic responses were observed in GABA_B_^+/-^ and wild type (GABA_B_^+/+^) mice but remained absent in GABA_B_^-/-^ mice, supporting the notion that GABA receptors regulate temperature homeostasis via the Z_tn_ [77]. Despite the established pentobarbital-GABA signaling link and the apparent relationship between GABA receptor agonism and T_b_ control, the evidence is presently too scant to classify pentobarbital as an anapyrexic agent.

### 6.6. (Chlor)promazine

Both chlorpromazine and promazine are drugs with antipsychotic effects. Chlorpromazine is used to treat schizophrenia, although promazine is the major metabolite found in chlorpromazine-treated schizophrenic patients and therefore constitutes the pharmacodynamically active compound. In rats chlorpromazine does not undergo metabolism as extensively as in in humans inasmuch as dechlorination results in less than 1/20 of the promazine plasma concentration in schizophrenic patients [79].

Chlorpromazine acts as an antagonist of dopamine-, serotonin-, adrenergic-, and muscarinic acetylcholine receptors. With respect to muscarinic acetylcholine receptors, chlorpromazine has an opposite effect of that of oxotremorine, which is a muscarinic acetylcholine receptor agonist. Considering that both (oxo)tremorine and chlorpromazine can induce hypothermia, it is unlikely that the induction of hypothermia is mediated solely by muscarinic receptors.

The effects of chlorpromazine on thermal effectors are inconsistent. On the one hand, chlorpromazine injection into the POAH of primates led to hypothermia with concomitant cutaneous vasodilation and respiratory acceleration (panting), which is suggestive of anapyrexia [80]. The intraperitoneal administration of chlorpromazine in rats resulted in a T_b_ reduction, characterized by inhibition of thermogenic shivering and piloerection and an increase in heat loss mechanisms such as augmented blood flow in the tail [81]. On the other hand, intracerebral injection of chlorpromazine into the POAH of rats produced a T_b_ increase [82, 83]. In mice, chlorpromazine was shown to substantially increase BAT activity [84]. This observation is particularly interesting since chlorpromazine has no sympathicomimetic properties, indicating possible central control.

Based on these contrasting reports, chlorpromazine does not unequivocally qualify as an anapyrexic agent. However, some of the pharmacodynamic features impart strong effects on the T_b_, making them an important focus of further research.

### 6.7. Insulin

Hypothermia is a common response to systemic insulin exposure as an anticipatory coping mechanism for an impending hypoglycemic state [85]. Hypoglycemia-induced hypothermia is not only prevalent in many small animals, but is also observed in humans following e.g., insulin shock therapy [86]. Infusion of 2-deoxyglucose, a metabolically inert glucose analogue, also results in lowering of T_b_ in humans, implying that hypoglycemia-induced hypothermia may comprise an evolutionary conserved mechanism across different species [87].

The main question, however, is whether hypothermia following (insulin-induced) hypoglycemia is anapyrexic in nature. In terms of anapyrexia, insulin should exert an inhibitory effect on thermogenesis in combination with stimulation of heat loss mechanisms. In ectothermic toads, for instance, induction of hypoglycemia via both insulin and 2-deoxyglucose is associated with a behavioral drift towards lower temperatures [88]. This behavioral pattern ultimately causes a reduction in T_b_ and constitutes one of the hallmarks of Z_tn_-mediated thermoregulation. Similarly, humans who become hypoglycemic at the expense of insulin or 2-deoxyglucose activate heat loss mechanisms such as sweating, vasodilation, and hyperventilation [87, 89, 90].

The major thermogenic effector BAT is under control of insulin, which stimulates its anabolic (endothermic) rather than its catabolic (thermogenic) activity [61]. It may, however, be the hypoglycemic state itself rather than the insulin that inhibits thermogenic signaling. Hypoglycemia-mediated inhibition of thermogenesis is in agreement with the finding that shivering is attenuated in cold-exposed human subjects who have become hypoglycemic [90].

It therefore appears that hypoglycemia, and not insulin per se, has anapyrexic potential. At this stage, however, hypoglycemia-induced anapyrexia is difficult to translate to a clinical application without understanding the underlying mechanism of action in the context of thermoregulation.

### 6.8. Acetaminophen

Acetaminophen is a well-known and widely used analgesic and antipyretic drug. Most research on the pharmacodynamics of acetaminophen is therefore mainly focused on the antipyretic properties. However, its role as an anapyrexic agent has been proposed, but remains controversial and inconclusive. Clinical studies have demonstrated a significant reduction in T_b_ following acetaminophen treatment of stroke, head trauma, and subarachnoid hemorrhage [91, 92]. However, these findings are inconsistent with other reports, in which acetaminophen treatment of stroke and administration after cardiac bypass surgery showed no significant effect on T_b_ [93, 94]. The latter reports do not preclude the possibility that higher dosages may exhibit a thermomodulatory effect, but the amount of data on acetaminophen-induced anapyrexic T_b_ reduction is too limited to draw solid conclusions at this point [95].

The thermoregulatory pharmacodynamics of acetaminophen remain elusive, although several advances in recent years have implicated the involvement of cyclooxygenase, peroxidase, nitric oxide synthase, canabinoid receptors, and serotonin receptors [95]. In an effort to elucidate the pharmacological mechanism of acetaminophen, a study in mice revealed that acetaminophen can reduce T_b_ from euthermic levels, which is in support of anapyrexic properties [96]. However, the underlying mechanisms remain obscure, with data suggesting an effect on anti-glutamate and anti-oxidant capacities rather than on thermoregulatory mechanisms [96]. Moreover, the T_b_-downmodulatory properties of acetaminophen may have been falsely ascribed in instances where the thermoregulatory system was already activated, or functionally compromised. These instances include underlying disease or clinical trauma (e.g., cancer or stroke, such as cited in the previous paragraph [91]) and bacterial and viral infections [97]. Finally, the hypothermic effect of acetaminophen has only been demonstrated in mice. Rats exposed to increasing acetaminophen dosages did not exhibit heat-avoiding behavior, indicating that no pharmacological modulation of the Z_tn_ had occurred [98]. Accordingly, the effect of acetaminophen on behavioral thermoregulation pleads against its classification as an anapyrexic agent, and it is unclear whether this class of drugs would induce hypothermia in a normal subject.

## 7. Concluding remarks

Anapyrexia has yet to gain widespread acceptance as a clinically functional state. Acknowledgement of its implementation through clinical pharmacology will largely depend on three factors: the development of efficient methods to adjust the Z_tn_ downward, the ability to accurately measure the boundaries of the Z_tn_, and the simultaneous use of external T_b_ control.

As indicated in the sections on the pharmacological agents above, there are various agents such as reserpine, (oxo)tremorine, and (chlor)promazine that exhibit specific aspects suggestive of an anapyrexic potential. However, due to the primary research focus on aspects other than anapyrexia, the anapyrexic potential of these agents requires further examination. Combinational therapy and translation to larger animal models constitute important steps towards the elucidation and optimization of the anapyrexic candidate drugs.

Ultimately, for clinical application, the simultaneous use of external T_b_ control alongside anapyrexic agents will be essential. Due to the high body surface:volume ratio of humans, passive lowering of T_b_ is too inefficient to support the depth of hypothermia that is dictated by the Z_tn_ under conditions of anapyrexia. Therefore, the advantage of anapyrexic agents lies in the facilitation of hypothermic therapy by optimizing thermal effectors (i.e., inhibition of thermogenic effectors and activation of heat loss effectors), thereby preventing the manifestation of Z_tn_-T_b_ mismatch-induced stressors during the induction of hypothermia.
